# Nutrition, Illness and Body Composition in Very Low Birth Weight Preterm Infants: Implications for Nutritional Management and Neurocognitive Outcomes

**DOI:** 10.3390/nu12010145

**Published:** 2020-01-04

**Authors:** Sara E. Ramel, Jacob Haapala, Jennifer Super, Christopher Boys, Ellen W. Demerath

**Affiliations:** 1 Department of Pediatrics, University of Minnesota, Minneapolis, MN 55454, USA; jsuper1@fairview.org (J.S.); boys0009@umn.edu (C.B.); 2 HealthPartners Institute, Minneapolis, MN 55425, USA; Jacob.L.Haapala@healthpartners.com; 3 Division of Epidemiology and Community Health, University of Minnesota, Minneapolis, MN 55454, USA; ewd@umn.edu

**Keywords:** body composition, preterm, neurodevelopment, nutrition, fat free mass

## Abstract

Preterm infants have altered body composition compared to term infants, which impacts both neurodevelopment and metabolic health, but whether increased dietary intake during hospitalization, independent of illness, may improve body composition is unknown. This prospective, longitudinal study (*n* = 103) measured fat-free mass (FFM) and percent body fat (%BF) at discharge and four months corrected age for prematurity (CA) in very low birth weight (VLBW) preterm infants. Markers of illness and macronutrient intakes (protein and caloric) were recorded. Bayley Scales of Infant Development-III (BSID) were administered at 12 and 24 months of age in a subset of these infants (*n* = 66 and *n* = 50 respectively). Body composition z-scores were calculated using recently developed reference curves. Linear regression was used to test the associations between clinical factors and body composition z-scores, as well as z-scores and BSID scores. Increased calories and protein received in the first week after birth and protein intake throughout hospitalization were associated with increased FFM z-scores at discharge, but not with %BF z-scores. After adjustment for both early acute and chronic illness, associations of nutrient intake with FFM z-score remained unchanged. FFM z-scores at discharge were positively associated with scores on the BSID at 12 and 24 months CA. In conclusion, increased energy and protein intakes both early in hospitalization and across its entire duration are associated with higher FFM at discharge, a key marker for organ growth and neurodevelopment in the VLBW neonate. Optimizing caloric intake, irrespective of illness is critical for enhancing body composition, and by extension, neurodevelopmental outcomes for preterm infants.

## 1. Introduction

Fat-free mass (FFM) gains are closely linked to brain development and are thus important growth parameters for the study of nutritional and physiological stressors on the preterm infant. Faster gains in FFM throughout hospitalization, as well as after hospital discharge, have been associated with improved neurocognitive outcomes, including higher cognitive and motor scores on standardized testing at 12 months corrected age for prematurity (CA), faster speed of processing in infancy and at preschool age and larger brain volumes at term CA [1,2,3,4,5]. Research into strategies that increase FFM gains are needed in order to optimize neurodevelopment without increasing metabolic risk.

Multiple factors have been shown to influence body composition in preterm infants, however many remain largely out of the neonatologist’s control. A few studies have shown that infants who received higher amounts of nutrients during hospitalization had improved FFM gains and neurodevelopment, however, it remains unclear if these associations are due to healthier infants receiving more nutrition and therefore having improved outcomes [2,6,7,8] Nutritional provision in the first week after birth appears to be particularly critical for both growth and neurodevelopment [2,6], however, nutrition provided throughout hospitalization has also been shown to be associated with linear growth out to at least two years of age in preterm infants [9].

Markers of critical illness have also been shown to influence short- and long-term growth and body composition amongst VLBW preterm infants [9,10]. We have previously reported on the prolonged negative influence of steroids, antibiotics/infection and days requiring oxygen (a surrogate for chronic lung disease) on linear growth out to two years CA [9].

It remains unclear whether the negative influence of illness on growth is independent of the decreased nutrient intake typically provided to ill infants due to concerns of tolerance or other factors inherent to illness such as growth hormone suppression, altered nutrient accretion due to inflammation, etc. Improved understanding of these relationships is critical to improving the nutritional care of this vulnerable population and in turn long-term outcomes.

In this study, we evaluated the influence of both illness and nutrient provision on later body composition to allow a better understanding of whether an increased nutrient provision is beneficial in terms of FFM gains for both healthy and sick preterm infants. In addition, we sought to determine whether early findings of improved neurodevelopment with increased FFM were sustained out to 24 months CA.

## 2. Methods

### 2.1. Study Population

One hundred and three infants were recruited from the Neonatal Intensive Care Unit (NICU) of the University of Minnesota Masonic Children’s Hospital from February 2012 to June 2016 into one of two observational studies with identical inclusion criteria. Inclusion criteria included birth weight <1500 g and appropriate for gestational age status (between the 10th and 90th percentile at birth on the Fenton Growth Curve) [11]. During the recruitment period, a total of 459 infants fewer than 1500 g at birth were admitted to the NICU. Of those excluded, 59 were small for gestational age (<10th percentile), 28 were large for gestational age (>90th percentile), six were older than one week on admission, 23 had anomalies, early clinical instability or died prior to consent, 132 declined participation, 39 parents were unable to consent, 18 transferred prior to consent, 26 were enrolled in a competing randomized trial, 20 had inability to follow-up and five had reasons unrecorded. The study protocols were approved by the University of Minnesota Institutional Review Board. Informed consent was obtained from the parents of the infants. This analysis utilizes data recorded from infants in both studies.

It was not possible to collect body composition measurements between 34–42 weeks PMA on 21 infants due to chronic lung disease requiring continuous respiratory support or transfer to a community hospital at an early gestational age. Eighty-one infants returned for follow-up at 4 months of age and 66 returned for neurodevelopmental testing at 12 months CA and 50 for neurodevelopmental testing at 24 months CA.

### 2.2. Inpatient Data Collection

Infants were followed daily while they were inpatient by the neonatal nutrition support service. Intake was recorded by the NICU dietician (J.S.). First-week intake was calculated by adding all calories and protein provided either enterally or parenterally from days 2–8 (DOL2–8). These dates were chosen as infants were born at different times on the first day of life and therefore the amount of nutrition provided on day of life one was variable based on the time of birth. The average daily energy and protein deficit was calculated as the average difference between 120 kcal/kg for energy and 4 g/kg for protein and the actual intake/kg of each up to 35 weeks PMA. Illness severity on postnatal days 12–14 were calculated using the score for neonatal acute physiology (SNAP-II), validated illness severity and mortality risk score for neonates, in which a higher score indicates increased severity of illness and a higher risk of mortality [12]. The number of inpatient days requiring positive pressure was recorded as a marker of prolonged critical illness as done in previous studies [8,9].

### 2.3. Anthropometrics and Body Composition

Body composition was measured prior to hospital discharge (between 34–42 weeks PMA) using air displacement plethysmography by the PEA POD. A detailed description of the PEA POD’s physical design, operating principles, validation in preterm infants, and measurement procedures have previously been detailed [13,14,15,16]. Briefly, the infant’s supine length was obtained in duplicate using a standard recumbent length measuring board to the nearest 0.1 cm. Using the integrated electronic scale on the PEA POD, infants were weighed without clothing or diapers to the nearest 0.0001 kg followed by a 2 min infant body volume measurement in the test chamber. Body density is then computed from body mass and volume. The PEA POD uses a constant fat mass density (0.9007 g/mL) and known age- and sex-specific fat-free mass density values [17] to calculate amounts of FFM and FM. Head circumference was measured in duplicate using a flexible cloth measuring tape to the nearest 0.1 cm. Body composition Z-scores were calculated using newly published preterm body composition reference curves for air displacement [18].

### 2.4. Neurodevelopment

Neurodevelopmental testing was performed utilizing the Bayley Scales of Infant Development-III that was performed by a pediatric neuropsychologist (C.B.) in our Neonatal Follow-up Clinic.

### 2.5. Statistical Analysis

Infant characteristics were described using means and standard deviations or frequencies. Multiple linear regressions were used to test the associations between individual nutrient intake measures (kcal and protein intakes during the first week, after birth and total deficits in kcal and protein intakes over the hospitalization period) and body composition z-scores (FFMZ and %BFZ) at two time points (hospital discharge and four months CA) in separate models. Multiple linear regression models were also used to test the association between body composition z scores (FFMZ and %BFZ) at both of the above time points and BSID scores at 12 and 24 months CA in separate models. For the first set of models (with body composition z-scores as the dependent variable), model 1 included covariates for study and gestational age at birth. Given that our primary hypothesis was that the relationship of nutrient intake on body composition was independent of early and chronic critical illness, we then included SNAP-II score at day of life 12–14 scores as a measure of early critical illness in model 2, and then added days requiring positive pressure as a statistical control for chronic critical illness in model 3. In the models with BSID scores as the dependent variable, model 1 included covariate adjustment for study and gestational age at birth. Given that IVH is a profound determinant of later neurodevelopment, model 2 added an indicator for IVH grade 2 or higher. Sensitivity analyses, including only participants who had complete data at all time points, were performed as a way of assessing whether any observed differences in the magnitude of the associations at different time points (discharge, four months, 12 months, and 24 months) were likely to be due to differential attrition. Associations were considered significant with *p* values < 0.05. Analyses were performed using SAS 9.4.

## 3. Results

Infant characteristics are detailed in Table 1. Infants were born at a mean GA of 27.8 weeks and had a mean birth weight of 1053 g. These infants were all born AGA with a mean BW z-score of −0.11. The majority of them underwent some growth restriction and had a weight z-score of −0.80 at hospital discharge. Those infants able to be measured at discharge had slightly higher mean birth weight (1089 g vs. 911 g *p* = 0.03) and higher mean gestational age at birth (28.1 weeks vs. 26.6 weeks *p* = 0.02) compared to those who were unable to be measured. There were no significant differences in birth weight or gestational age at birth between those included and not included at any other visit time (four, 12 and 24 months CA).

First-week caloric and protein intakes were positively associated with FFMZ at discharge in the minimally adjusted models, and these associations remained statistically significant after the addition of illness variables (SNAP score and days requiring positive pressure) (Table 2). Average daily caloric and protein deficits over the course of hospitalization were also associated with FFMZ at discharge in minimally adjusted models. The association between average daily caloric intake and FFMZ diminished with adjustment for total days requiring positive pressure. Neither first-week caloric and protein intakes nor total kcal and protein deficits were associated with %BFZ (Table 2).

First-week caloric intake and the average daily caloric deficit remained negatively associated with FFMZ at four months of age (Table 3). These associations remained statistically significant after adjustment for both early and chronic critical illness. In contrast, the observed associations of first-week protein intake and total protein deficit with FFMZ at discharge were no longer present at four months CA regardless of illness adjustment (Table 3).

With regard to the relationship of body composition z-scores to infant neurodevelopment, FFMZ at discharge, but not %BFZ was positively associated with both cognitive and motor scores on the BSID at 12 months CA (*p* ≤ 0.04 for both), and with motor scores on the BSID at 24 months CA (*p* = 0.04). For each of these associations, there was an approximately 3-point improvement on the Bayley for each increase in FFM z-score. Neither FFMZ nor %BFZ at 4 months CA were associated with any score on the BSID at either 12 or 24 months CA (Table 4).

Sensitivity analyses restricted to individuals with complete data showed a similar magnitude of association for the relationship of nutrient intake to body composition z scores and between those z scores and BSID scores as they did in the entire cohort (data not shown).

## 4. Discussion

We prospectively gathered detailed data on nutrient intake and markers of critical illness and utilized air displacement plethysmography to measure body composition at hospital discharge and four months CA to investigate clinical factors associated with improved FFM in early infancy (discharge and four months CA). In addition, we performed standardized neurodevelopmental testing at multiple time points to establish whether associations between FFM and neurodevelopment extend beyond 12 months CA. The importance of quality of growth as a marker of long-term outcomes is becoming increasingly recognized. Yet, it is still unknown how to effectively modify FFM gains in preterm infants. In this group of very low birth weight preterm infants, we found that increased energy intake in the first week after birth as well as throughout hospitalization is associated with increased FFM throughout early infancy independent of early and chronic critical illness. In turn, FFM z-scores, but not %BF z-scores calculated using newly released reference curves [18], were associated with improved scores on the BSID at 12 and 24 months CA. Increased nutrient provision was not associated with %BF z-scores at either time point showing that the benefits of increased nutrition on fat-free mass do not come at the cost of increased adiposity. These findings suggest that nutrition protocols that aim to provide enhanced nutrition to all infants, regardless of illness status, and starting immediately after birth will likely have a positive influence on the quality of growth as well as neurodevelopmental outcomes.

There are several small and/or retrospective studies that have documented associations between markers of critical illness and long-term growth outcomes. Increased days receiving antibiotics or steroids and days requiring positive pressure ventilation have been associated with decreased linear growth out to two years of age [9]. In addition, increased illness scores on the first day of life are associated with decreased amounts of FFM present at four months of age [10]. When comparing preterm infants with bronchopulmonary dysplasia (BPD) with healthy term infants, those with BPD have slower gains in weight, length, FFM and FM out to at least one year of age [19]. In a study utilizing whole-body magnetic resonance imaging to measure body composition, Uthaya et al. found that illness severity was the primary predictor of abdominal adiposity [20]. None of these studies investigated the mechanisms behind these relationships, specifically whether the poor growth was related to decreased nutrient provision to those infants who were sicker.

One of the largest studies specifically looking at both nutritional and non-nutritional factors influencing body composition involved 141 preterm infants born in France. The infants involved in this study were born prior to 35 weeks with a mean gestational age at birth of 31.5 weeks [21], representing a likely much more mature, healthy and varied group than those presented in our current study (mean GA 27.8 weeks; range 22.1–32.4 weeks). Simon et al. collected nutritional data on a few selected days throughout hospital stay (5, 10, and 21) rather than cumulative intake, and also looked at early growth trajectories, antenatal steroid treatment and days requiring respiratory support. They found that gestational age at birth, early growth trajectories, and higher protein-energy ratio provided on days 10 and 21 of life were associated with increased FFM gains. They found no association with respiratory support. In the present study, we found that higher SNAP/illness scores and days requiring positive pressure and steroids were negatively associated with FFM at discharge and associations with positive pressure days remained at four months. We expanded the findings of the Simon study through the examination of the impact of cumulative nutrient intake and illness together in a more immature group of infants.

While it is accepted that more critically ill preterm infants have poorer growth when compared to healthier infants born at similar ages, it remains unclear how much of this is due to restricted intake provided to this subset of infants versus a direct effect of inflammation on nutrient accretion. In a mediation analysis of more than 1000 preterm born infants, Eherenkranz et al. found that infants who were less critically ill had a higher nutrient intake, increased weight gain, and better long-term outcomes and that these relationships were largely mediated by first-week nutrient intake [8]. In this study they used days on positive pressure as a surrogate for critical illness as well. Our analysis confirmed and extended these findings to show that this is not only true for weight gain but more specifically FFM gains. In addition, we looked not only at first-week intake but also cumulative caloric intake, which also positively influenced FFM gains despite critical illness. We are not aware of other studies investigating the complex interplay of early nutrition, illness and body composition among preterm infants.

Our study also confirmed the findings of several other studies related to the positive relationship between FFM gains during hospitalization and long-term neurodevelopment. These previous studies also found that gains in FFM, but not FM, prior to hospital discharge, during a time of rapid brain growth and development, were associated with faster speed of brain processing in infancy and at preschool age [1,4], higher standardized test scores at 12 months of age [2], higher scores on the ages and stages questionnaire at two years of age [22] and larger brain volumes [5]. The current analysis extends previous findings by confirming that the relationships between higher amounts of FFM and higher standardized test scores persist out to 24 months of age. However, the current study differed from some of the previous studies in that gains in FFM after term were no longer associated with neurodevelopmental outcomes [1,3,4], suggesting a potentially smaller window of time to intervene with regards to optimizing FFM gains with the goal of improving later neurodevelopment.

Our current study utilized newly published reference curves for infant body composition [18], showing that FFM z-scores at term corrected age that are closer to those of infants born healthy at term, may lead to improved neurodevelopmental outcomes, similar to the AAP recommendation [23]. This study is one of the largest prospective longitudinal studies with specific data collected on body composition and neurodevelopment at multiple time points. In addition, it has detailed nutrient data and illness data collected prospectively throughout hospitalization.

Unfortunately, there was significant participant drop-out over time leading to smaller sample sizes at 12 and 24 months CA. A sample size calculation was not made in advance in this observational study; therefore, there is a possibility of type I error. Further, interpretation of the results should consider that an adjustment for multiple comparisons was not made. Due to the observational design, the causality of the associations cannot be inferred. Randomized nutrition intervention trials are needed to determine whether an enhanced nutrient provision is both feasible and beneficial when given to critically ill inflamed preterm infants.

## 5. Conclusions

In conclusion, early nutrition has a lasting impact on growth that persists beyond the period of provision and is beneficial with regards to FFM gains, without increasing FM gains, regardless of critical illness. Results from this study could inform future randomized trials and neonatologists as they make decisions regarding the nutrient provision and in turn improve long-term neurodevelopment and overall health outcomes for critically ill preterm neonates.

## Figures and Tables

**Table 1:** Description of 103 VLBW preterm infants.

Variable		*n* (%)	Mean (SD)	Median	Min–Max
**Inpatient**					
Study	Amplatz	56 (54.4)			
	March of Dimes	47 (45.6)			
Sex	Female	49 (47.6)			
	Male	54 (52.4)			
GA at birth (weeks)		103	27.8 (2.4)	28.3	22.1–32.4
Weight at birth (g)		103	1053 (300)	1090	408–1730
Weight z-score at birth		103	−0.11 (0.69)	−0.15	−1.63–1.20
Weight z-score at discharge		81	−0.80 (0.87)	−0.78	−3.94–1.50
Total kcal/kg, week 1		103	669 (94)	675	447–859
Total protein (g)/kg, week 1		103	25.2 (3.2)	25.8	15.3–31.8
Average daily kcal/kg deficit, 35 weeks		103	9.0 (7.1)	7.6	−5.6–29.1
Average daily protein (g)/kg deficit, 35 weeks		103	0.23 (0.31)	0.22	−0.52–0.96
Total positive pressure days, 35 weeks		103	20.9 (23.9)	10.0	0.0–89.0
IVH, Grade 2 or higher	No	85 (82.5)			
	Yes	18 (17.5)			
SNAP score, day 12–14	0	67 (65.7)			
	1 to 16	35 (34.3)			
**Discharge**					
PCA (weeks), discharge		82	37.4 (2.3)	36.8	34.0–41.9
FM z-score, discharge		82	1.8 (1.2)	1.8	−0.8–5.4
FFM z-score, discharge		82	−1.4 (1.2)	−1.6	−5.0–1.4
%BF z-score, discharge		82	2.3 (1.1)	2.3	−0.3–4.9
**4 Months Corrected Age**					
PCA (wks), 4 month CA visit		81	57.7 (1.7)	57.7	54.0–63.0
FM z-score, 4 months		76	−1.1 (1.3)	−0.9	−4.6–2.0
FFM z-score, 4 months		76	0.06 (1.52)	0.31	−3.39–3.50
%BF z-score, 4 months		76	−1.2 (1.2)	−1.1	−4.6–1.4
**12 Months Corrected Age**					
Bayley Cognition, 12 months		66	101 (14)	100	60–135
Bayley Language, 12 months		65	87.0 (13.7)	86.0	50–121
Bayley Motor, 12 months		66	90.4 (14.5)	91.0	58–124
**24 Months Corrected Age**					
Bayley Cognition, 24 months		50	95.0 (15.4)	95.0	55–145
Bayley Language, 24 months		46	91.7 (17.9)	94.0	47–147
Bayley Motor, 24 months		48	90.0 (16.9)	92.5	52–115

**Table 2:** Associations between Nutrient intake, Early and chronic illness and Body composition z-scores at Hospital Discharge among VLBW preterm infants.

		Model 1		Model 2		Model 3	
	*n*	ß (SE)	*p* Value	ß (SE)	*p* Value	ß (SE)	*p* Value
**FFM Z-Score, Discharge**							
Total kcal/kg, week 1	82	0.0074 (0.0016)	<0.0001	0.0060 (0.0018)	0.001	0.0054 (0.0019)	0.005
Total protein (g)/kg, week 1	82	0.1256 (0.0386)	0.002	0.1126 (0.0365)	0.003	0.1012 (0.0375)	0.009
Average daily kcal/kg deficit, 35 weeks	82	−0.0758 (0.0214)	0.0007	−0.0523 (0.0235)	0.029	−0.0403 (0.0268)	0.14
Average daily protein (g)/kg deficit, 35 weeks	82	−1.1400 (0.4467)	0.013	−1.1606 (0.4144)	0.006	−1.0490 (0.4187)	0.014
Total positive pressure days, 35 weeks	82	−0.0348 (0.0100)	0.0009	−0.0221 (0.0118)	0.06		
**%BF Z-Score, Discharge**							
Total kcal/kg, week 1	82	0.0018 (0.0017)	0.29	−0.0001 (0.0019)	0.96	−0.0003 (0.0020)	0.88
Total protein (g)/kg, week 1	82	0.0388 (0.0382)	0.31	0.0300 (0.0376)	0.43	0.0289 (0.0390)	0.46
Average daily kcal/kg deficit, 35 weeks	82	−0.0229 (0.0214)	0.29	−0.0013 (0.0236)	0.95	0.0029 (0.0271)	0.92
Average daily protein (g)/kg deficit, 35 weeks	82	−0.5499 (0.4306)	0.21	−0.5630 (0.4191)	0.18	−0.5573 (0.4292)	0.20
Total positive pressure days, 35 weeks	82	−0.0149 (0.0099)	0.14	−0.0037 (0.0117)	0.75		

Each nutritional and illness-related predictor of FFMZ and %BFZ was tested in a separate model; Model 1 adjusted for study and GA at birth; Model 2 adjusted for study, GA at birth, and SNAP score at day 12–14; Model 3 adjusted for study, GA at birth, SNAP score at day 12–14, and days requiring positive pressure.

**Table 3:** Associations between Nutrient intake, Early and Chronic illness and Body composition z-scores at four months CA among VLBW preterm infants.

		Model 1		Model 2		Model 3	
	*n*	ß (SE)	*p* Value	ß (SE)	*p* Value	ß (SE)	*p* Value
**FFM Z-Score, 4 Months**							
Total kcal/kg, week 1	75	0.0078 (0.0024)	0.002	0.0076 (0.0027)	0.005	0.0062 (0.0028)	0.029
Total protein (g)/kg, week 1	75	0.0534 (0.0550)	0.34	0.0505 (0.0546)	0.36	0.0089 (0.0565)	0.88
Average daily kcal/kg deficit, 35 weeks	75	−0.0927 (0.0278)	0.001	−0.0885 (0.0302)	0.004	−0.0723 (0.0316)	0.025
Average daily protein (g)/kg deficit, 35 weeks	75	−0.7238 (0.6711)	0.28	−0.7452 (0.6652)	0.27	−0.5685 (0.6512)	0.39
Total positive pressure days, 35 weeks	75	−0.0408 (0.0143)	0.006	−0.0394 (0.0165)	0.020		
**%BF Z-Score, 4 Months**							
Total kcal/kg, week 1	75	0.0019 (0.0022)	0.37	−0.0000 (0.0024)	0.99	−0.0013 (0.0025)	0.59
Total protein (g)/kg, week 1	75	0.0068 (0.0472)	0.89	0.0032 (0.0460)	0.95	−0.0228 (0.0484)	0.64
Average daily kcal/kg deficit, 35 weeks	75	−0.0040 (0.0255)	0.88	0.0185 (0.0267)	0.49	0.0354 (0.0278)	0.21
Average daily protein (g)/kg deficit, 35 weeks	75	0.6698 (0.5713)	0.24	0.6443 (0.5572)	0.25	0.7565 (0.5540)	0.18
Total positive pressure days, 35 weeks	75	−0.0297 (0.0124)	0.019	−0.0216 (0.0142)	0.13		

Each nutritional and illness related predictor of FFMZ and %BFZ was tested in a separate model. Model 1 adjusted for study and GA at birth. Model 2 adjusted for study, GA at birth, and SNAP score at day 12–14. Model 3 adjusted for study, GA at birth, SNAP score at day 12–14, and days requiring positive pressure.

**Table 4:** Associations between Body composition z-scores and Neurodevelopmental outcomes (BSID scores at 12 and 24 months CA).

		Model 1		Model 2	
	*n*	ß (SE)	*p* Value	ß (SE)	*p* Value
**Bayley Cognition, 12 Months**					
FFM z-score, discharge	53	3.2899 (1.3978)	0.023	2.9114 (1.3886)	0.041
%BF z-score, discharge	53	−0.3243 (1.7055)	0.85	−0.2069 (1.6579)	0.90
FFM z-score, 4 months	57	0.9511 (1.1376)	0.41	0.6384 (1.1032)	0.57
%BF z-score, 4 months	57	1.0280 (1.4965)	0.50	1.3095 (1.4349)	0.37
**Bayley Language, 12 Months**					
FFM z-score, discharge	52	1.8446 (1.3940)	0.19	1.7349 (1.4228)	0.23
%BF z-score, discharge	52	−0.4566 (1.6462)	0.78	−0.4240 (1.6563)	0.80
FFM z-score, 4 months	56	−1.1535 (1.0893)	0.29	−1.2751 (1.0971)	0.25
%BF z-score, 4 months	56	0.5436 (1.4245)	0.70	0.6521 (1.4333)	0.65
**Bayley Motor, 12 Months**					
FFM z-score, discharge	53	3.9306 (1.4443)	0.009	3.5447 (1.4359)	0.017
%BF z-score, discharge	53	−0.2697 (1.7924)	0.88	−0.1461 (1.7421)	0.93
FFM z-score, 4 months	57	1.2866 (1.1265)	0.26	0.9613 (1.0867)	0.38
%BF z-score, 4 months	57	−0.0048 (1.4969)	0.99	0.2849 (1.4302)	0.84
**Bayley Cognition, 24 Months**					
FFM z-score, discharge	42	2.8657 (1.4491)	0.06	2.1410 (1.3452)	0.12
%BF z-score, discharge	42	0.5661 (1.7093)	0.74	0.7323 (1.5336)	0.64
FFM z-score, 4 months	45	−0.7448 (1.5205)	0.63	−1.1685 (1.3315)	0.39
%BF z-score, 4 months	45	−0.0076 (2.1207)	0.99	1.2707 (1.8840)	0.50
**Bayley Language, 24 Months**					
FFM z-score, discharge	38	3.5723 (2.0776)	0.09	2.7798 (2.1356)	0.20
%BF z-score, discharge	38	2.4214 (2.3715)	0.31	2.2233 (2.3107)	0.34
FFM z-score, 4 months	42	−0.3577 (1.7575)	0.84	−0.8526 (1.6807)	0.61
%BF z-score, 4 months	42	2.1388 (2.4787)	0.39	2.9154 (2.3546)	0.22
**Bayley Motor, 24 Months**					
FFM z-score, discharge	41	4.4815 (1.8473)	0.020	3.4026 (1.6495)	0.046
%BF z-score, discharge	41	1.9762 (2.2422)	0.38	2.1937 (1.9180)	0.26
FFM z-score, 4 months	43	0.7622 (1.6217)	0.64	0.0541 (1.3605)	0.97
%BF z-score, 4 months	43	−0.9982 (2.2731)	0.66	0.3561 (1.9175)	0.85

Each body composition z-score related predictor of BSID scores was tested in a separate model. Model 1 adjusted for study, GA at birth. Model 2 adjusted for study, GA at birth, and IVH indicator (Grade 2 or higher).

## Abbreviations

VLBW	Very low birth weight
CA	Corrected age for prematurity
FFM	Fat-free mass
FM	Fat mass
%BF	Percent body fat
BSID	Bayley Scales of Infant Development-III
FFMZ	Fat-free mass z-score
%BFZ	Percent body fat z-score
NICU	Neonatal intensive Care Unit
PMA	Post menstrual age
SNAP	Score for Neonatal Acute Physiology
IVH	Interventricular hemorrhage
